# Human milk unmetabolized folic acid is increased following supplementation with synthetic folic acid as compared to (6*S*)-5-methyltetrahydrofolic acid

**DOI:** 10.1038/s41598-023-38224-4

**Published:** 2023-07-12

**Authors:** Kelsey M. Cochrane, Rajavel Elango, Angela M. Devlin, Jennifer A. Hutcheon, Crystal D. Karakochuk

**Affiliations:** 1 Food, Nutrition and Health, Faculty of Land and Food Systems, The University of British Columbia, 2205 East Mall, Vancouver, BC V6T 1Z4 Canada; 2 BC Children’s Hospital Research Institute, Healthy Starts, Vancouver, Canada; 3 Department of Paediatrics, Faculty of Medicine, The University of British Columbia, Vancouver, Canada; 4 Population and Public Health, Faculty of Medicine, The University of British Columbia, Vancouver, Canada; 5 Department of Obstetrics and Gynaecology, Faculty of Medicine, The University of British Columbia, Vancouver, Canada

**Keywords:** Paediatric research, Nutrition, Clinical trials

## Abstract

Folic acid supplementation is recommended perinatally, but may increase unmetabolized folic acid (UMFA) in human milk; this is concerning as it is an inactive form which may be less bioavailable for the infant. “Natural” (6*S*)-5-methyltetrahydrofolic acid [(6*S*)-5-MTHF] is available as an alternative to folic acid, and may prevent the accumulation of UMFA in human milk. Pregnant women (*n* = 60) were enrolled at 8–21 weeks of gestation and randomized to 0.6 mg/day folic acid or (6*S*)-5-MTHF. At ~ 1-week postpartum, participants provided a human milk specimen. Total human milk folate (nmol/L) and concentrations of UMFA (nmol/L) were quantified via LC–MS/MS. Differences between groups were evaluated using multivariable quantile/linear regression, adjusting for dietary folate, weeks supplementing, and milk collection methods. No significant difference in total milk folate was found; however, the median milk UMFA concentration was 11 nmol/L higher in those receiving folic acid versus (6*S*)-5-MTHF (95% CI = 6.4–17 nmol/L), with UMFA representing 28% and 2% of total milk folate. In conclusion, the form of supplemental folate had markedly differential effects on the human milk folate profile, with folic acid increasing the mean proportion of milk UMFA by ~ 14-fold. Investigation of whether increased UMFA impacts folate-related metabolism and infant health outcomes is required.

## Introduction

Human milk provides optimal nutrition for infants and has many established health benefits for both the mother and child^1,2^. Short term benefits of human milk for infants are clear (reducing the risk of all-cause mortality and infectious diseases), and increasing evidence suggests long-lasting protection against chronic diseases and increased intelligence in adulthood^3–5^. Both biological and environmental factors contribute to human milk composition^2,6^. Consumption of prenatal multivitamins (providing 0.4 mg/day folic acid) is recommended preconceptionally until the end of lactation to support healthy development of the fetus and infant (via human milk), while meeting maternal nutrient needs^7,8^. Understanding how modifiable factors, such as maternal micronutrient intake, affect human milk composition is imperative to supporting optimal breastfeeding practices and thus, infant health.

Exclusively breastfed infants rely on human milk to meet their micronutrient needs (with the exception of vitamin D drops)^6,9^. Adequate folate is critical during periods of growth given its role in one carbon (1C) metabolism, cellular proliferation, and methylation of DNA, RNA, and proteins^10^. Naturally occurring folates are mainly reduced and are present in numerous chemically-related forms; 5-methyltetrahydrofolate (5-MTHF) is widely reported as the predominant form in human milk^11,12^, constituting ~ 50%^13^ to ~ 70%^14^ of total folate in mature milk. As folate circulates in plasma predominantly as 5-MTHF (representing > 90% of plasma folate^15^), it is proposed that folates may be interconverted within the mammary epithelium^16–18^. Most studies suggest that total human milk folate is not substantially affected by maternal diet unless severe deficiency occurs^11,14,19,20^. However, the impact of supplemental folic acid, an oxidized, synthetic form of folate, on the human milk folate profile is unclear^21^.

Upon ingestion, folic acid must be reduced to the metabolically active form (5-MTHF); however, the capacity for intestinal reduction is limited, resulting in unmetabolized folic acid (UMFA) entering systemic circulation^22,23^. UMFA is widely detected among adults^24^ and pregnant women in the United States and Canada^25^; UMFA is an inactive form of folate, but its impact on folate-related metabolism and infant health remains unclear. The developmental origins of health and disease hypothesis suggests that susceptibility to future health and disease risk may be partially mediated by exposures in utero and early life^26^; such exposures may include elevated concentrations of folate or UMFA, leading to perturbations in 1C metabolism and impacting fetal programming via epigenetic mechanisms, altered gene expression, and changes in cellular function^21,27,28^.

Folate is transported across the mammary epithelium via the folate binding protein (FBP), folate receptor alpha (FRα)^29^. The affinity of FRα for oxidized folates (UMFA) is 6–tenfold greater than for reduced forms, potentially leading to the preferential uptake of UMFA into human milk^29,30^. Exact transport mechanisms and the correlation of maternal plasma UMFA with the human milk folate profile remains poorly described^16,18,19^. However, as compared to those supplementing with < 0.4 mg/day folic acid in the Maternal-Infant Research on Environmental Chemicals cohort (MIREC; *n* = 561), supplementation with > 0.4 mg/day increased the proportion of UMFA as part of total milk folate by twofold^13^. In those consuming > 0.4 mg/day folic acid, the human milk folate profile shifted so that UMFA became the predominant folate species (representing 50% of total human milk folate)^13^.

Prenatal vitamins containing an analogue of natural folate [(6*S*)-5-methyltetrahydrofolic acid; (6*S*)-5-MTHF] are widely available^31^, but evidence regarding the effect of supplemental folic acid versus (6*S*)-5-MTHF on the human milk folate profile is scarce. Houghton et al.^19^ randomized lactating women to 0.416 mg/day (6*S*)-5-MTHF (*n* = 21) or a placebo (*n* = 20) starting at 1 week postpartum, and included a folic acid (0.4 mg/day) reference group (*n* = 16). Detectable UMFA was reported in 96% of human milk specimens, with no differences between groups^19^. However, findings from the MIREC cohort suggest that the impact of folic acid on the human milk folate profile is dose-dependent^13^. Thus, the lack of differences reported by Houghton et al. may have been attributed to the lower dose (0.4 mg/day)^19^; commercial prenatal vitamins in North America contain doses of folic acid > 0.4 mg (generally 0.6–1.0 mg)^31,32^.

This study was a secondary analysis in a randomized clinical trial and aimed to evaluate the impact of supplementation with 0.6 mg/day folic acid or an equimolar dose (0.625 mg/day) of (6*S*)-5-MTHF on the human milk folate profile. We hypothesized that folic acid supplementation would elicit higher UMFA in human milk (in absolute concentrations and proportionally) than (6*S*)-5-MTHF.

## Results

### Characteristics of the study population and human milk collection

**Figure 1:**
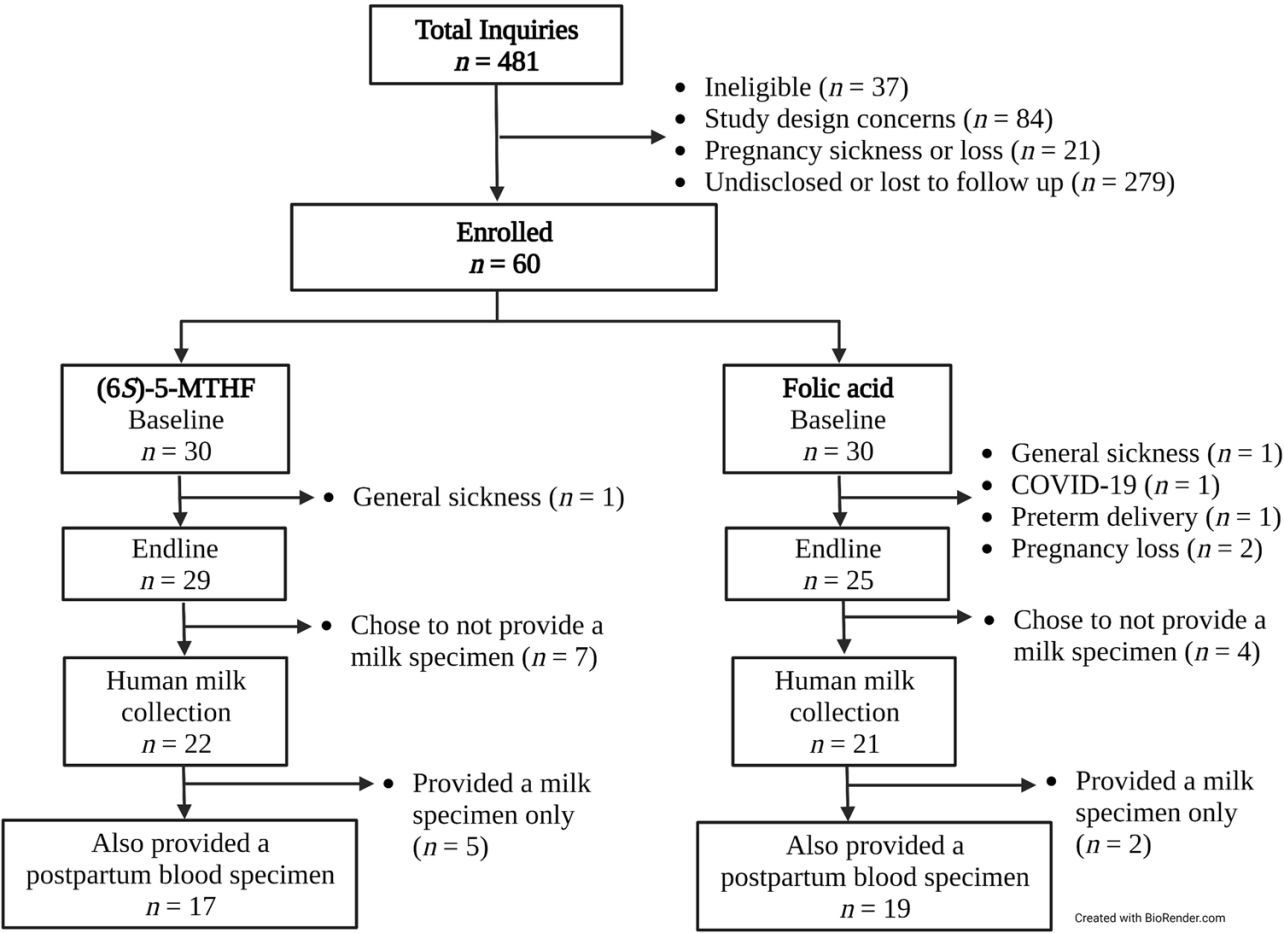
Participant flow diagram. Baseline visits took place at 8–21 weeks’ gestation. Endline visits took place 16-weeks after the baseline visit (24–38 week’s gestation). Human milk and postpartum blood specimens were collected at ~ 1-week postpartum.

A total of *n* = 43 participants provided a human milk specimen; of those, 84% (*n* = 36) also provided a postpartum blood specimen (see Fig. 1). Participant characteristics are presented in Table 1 Most participants (*n* = 39; 91%) were full term at delivery. The mean ± SD total weeks of supplementing was 24 ± 4 weeks. The median (IQR) daily supplementation rate at the human milk collection was 98% (94 to 100%).

**Table 1:** Characteristics of participants who provided a human milk sample at ~ 1-week postpartum following supplementation with (6*S*)-5-MTHF or folic acid throughout pregnancy (Vancouver, Canada, 2019–2021).

	(6*S*)-5-MTHF (n = 22)	Folic acid (n = 21)
Age, years	32 ± 3	33 ± 3.2
Ethnicity, n (%)		
European	11 (50)	12 (57)
South, East, and Southeast Asian	5 (22)	5 (24)
Hispanic/Latino	4 (18)	2 (9.5)
Middle eastern	1 (5)	0 (0)
Mixed ethnicity	1 (5)	2 (9.5)
Education, n (%)		
Highschool	1 (5)	0 (0)
College	4 (18)	1 (5)
Undergraduate	8 (36)	12 (57)
Graduate	9 (41)	8 (38)
^a^Household income per year, CAD$, n (%)		
< 20,000	1 (5)	0 (0)
20,000–50,000	1 (5)	3 (15)
50,000–100,000	6 (27)	7 (35)
> 100,000	14 (63)	10 (50)
Nulliparous, n (%)	18 (82)	12 (57)
^b^Dietary folate intake, mg DFEs/day	0.52 ± 0.17	0.46 ± 0.15
^c^Total weeks supplementing	24 ± 3.6	24 ± 5.2
Weeks of gestation at delivery	39 ± 1.5	39 ± 1.6
^d^Full term (≥ 37 wks) at delivery, n (%)	20 (91)	19 (90)

Results are mean ± SD unless otherwise noted.

*DFEs* Dietary folate equivalents.

^a^Missing *n* = 1 household income in the folic acid group (participant chose not to disclose).

^b^DFEs from food sources only.

^c^Total weeks supplementing from baseline (8–21 weeks of gestation) to the milk collection (~ 1-week postpartum).

^d^Those who delivered prematurely were between 35–36 weeks of gestation.

The mean ± SD day postpartum at the human milk collection was day 7 ± 3, and frozen milk samples were picked up within 4 days for 93% (*n* = 40) of participants; for *n* = 3 participants, pick-up occurred after 5 days (*n* = 2) and 10 days (*n* = 1). The blood specimen collection (as applicable) took place upon human milk pick-up (except for 1 participant; due to COVID-19 pandemic countermeasures, the blood draw occurred 10 days after a contactless human milk pick-up). Most participants collected the human milk specimen from the right breast (88%) and between 1300–1450 h (84%). Most participants fully expressed their breast upon milk collection (67%) and most used an electric pump (77%). However, rates of fully expressing were 50% (*n* = 11) and 86% (*n* = 18) in the (6*S*)-5-MTHF and folic acid groups, respectively. Median (IQR) hours of maternal folate supplementation prior to the human milk and blood collections, respectively, were 2.25 h (2 to 3 h) and 2 h (2 to 2.5 h); *n* = 2 did not record the timing of supplementation before the milk collection).

### Human milk folate profile at 1 week postpartum

**Figure 2:**
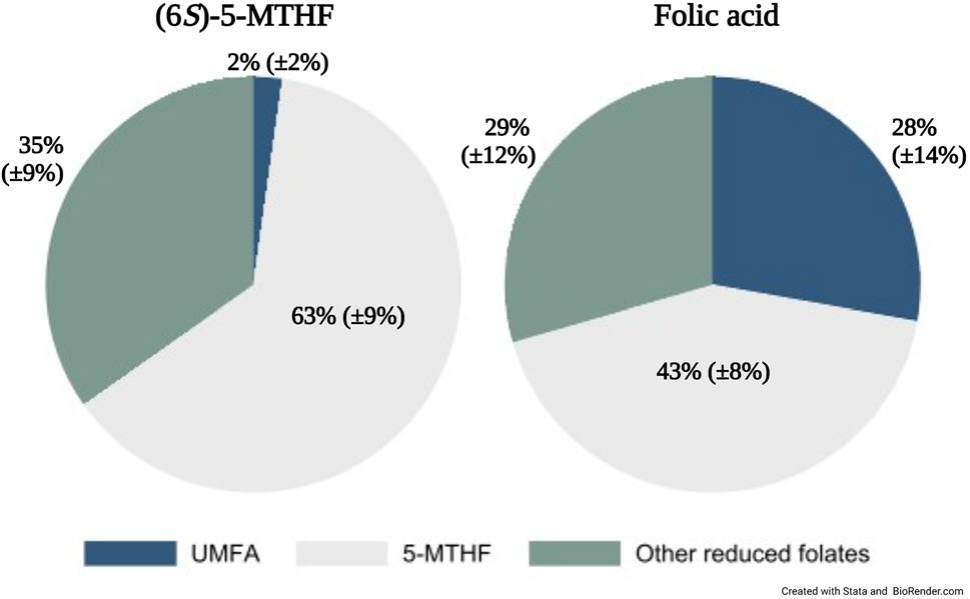
Human milk folate profile among 43 participants supplemented with (6*S*)-5-MTHF (n = 22) or folic acid (n = 21). The mean ± SD for the proportion of each form is presented. “5-MTHF” includes: 5-MTHF and MeFox (an oxidation product of 5-MTHF); “Other reduced folates” include: THF, 5,10-methenyl-THF, and 5-formyl-THF. Abbreviations: *5-MTH*F 5-methyltetrahydrofolate; *THF* Tetrahydrofolate; *UMFA* Unmetabolized folic acid.

Biochemical outcomes are summarized in Table 2 (see Supplementary Table 1 for full model outputs). Mean levels of total milk folate were not significantly different between groups, with a high degree of variability in the crude estimates. Further, no significant differences were found between any of the reduced folate forms in the human milk specimens (5-MTHF, THF, 5,10-methylTHF, 5-formylTHF) or MeFox. While no statistically significant difference was observed (*p* = 0.10), concentrations of human milk 5-MTHF trended higher following supplementation with (6*S*)-5-MTHF versus folic acid, with a median (IQR) of 29 (17, 36 nmol/L) and 21 (18, 27 nmol/L), respectively. Conversely, the median concentration of human milk UMFA in those supplemented with folic acid was 11 nmol/L (adjusted 95% CI = 6.4–17 nmol/L) higher than in those consuming (6*S*)-5-MTHF. Differences in the human milk folate profile is further visualized in Fig. 2; mean ± SD proportion of milk UMFA as part of total milk folate was 2% (± 2%) and 28% (± 14%) in (6*S*)-5-MTHF and folic acid groups, respectively.

**Table 2:** Effect of supplemental folate form on the human milk folate profile among women supplemented with (6*S*)-5-MTHF or folic acid during pregnancy and lactation (Vancouver, Canada, 2019–2021).

	(6*S*)-5-MTHF crude concentrations	Folic acid crude concentrations	Crude difference (95% CI)	^a^Adjusted difference (95% CI)
Human milk (*n* = 43)				
Participants, *n*	22	21	–	–
UMFA (nmol/L)	0.6 (0.3, 1.1)	12 (9.9, 18)	11 (6.5, 16)	11 (6.4, 17)
5-MTHF (nmol/L)	29 (17, 36)	21 (18, 27)	− 7.2 (−16, 1.5)	− 9 (−20, 1.8)
THF (nmol/L)	13 (7.8, 20)	12 (8.0, 24)	− 1.3 (−11, 8)	− 0.8 (−9.4, 7.9)
5,10-methenylTHF (nmol/L)	2.1 (1.2, 2.6)	1.6 (1.2, 3.3)	− 0.5 (−1.7, 0.8)	− 0.6 (−1.8, 0.6)
5-formylTHF (nmol/L)	0.7 (0.5, 1.2)	0.9 (0.5, 1.1)	0.1 (−0.4, 0.6)	0.3 (−0.2, 0.8)
MeFox (nmol/L)	0.6 (0.5, 0.8)	0.6 (0.5, 0.8)	0.02 (−0.2, 0.2)	0.07 (−0.1, 0.3)
^b^Total folate (nmol/L)	45 (29, 63); 47 ± 20	53 (40, 70); 61 ± 28	13 (−1.8, 28)	13 (−2.9, 29)
Maternal plasma (*n* = 36)				
Participants, *n*	17	19	–	–
UMFA (nmol/L)	0.8 (0.7, 1.2)	13 (7, 23)	12 (5.9, 19)	–
5-MTHF (nmol/L)	72 (64, 96); 74 ± 27	68 (53, 84); 71 ± 24	− 2.6 (−20, 15)	–

Results are median (IQR); mean ± SD is also presented if normally distributed.

*5-MTHF* 5-methyltetrahydrofolate; *THF* Tetrahydrofolate; *UMFA* Unmetabolized folic acid.

^a^Milk folate forms and total folate adjusted for: supplemental folate form ([6*S*]-5-MTHF as the reference group), milk expression (partial expression as the reference group), total weeks of supplementing, dietary folate intake (mg dietary folate equivalents/d).

^b^Total folate = sum of all forms.

### Association of UMFA in maternal plasma and human milk

**Figure 3:**
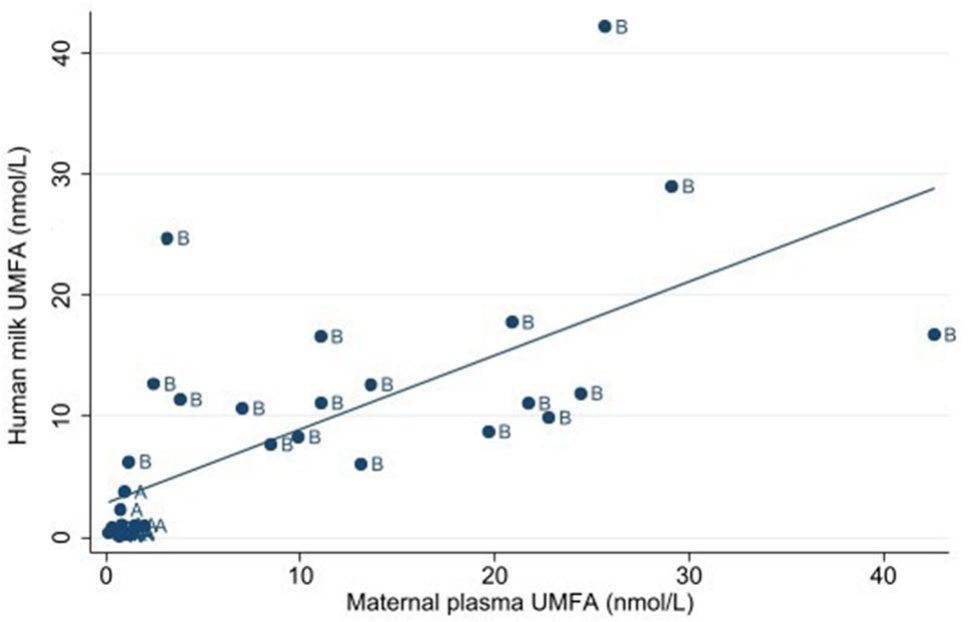
Association of maternal plasma UMFA and human milk UMFA among 36 participants at 1 week postpartum. A two-way linear prediction scatterplot. **“A”:** (6*S*)-5-MTHF (n = 17) and **“B”:** folic acid (n = 19). Abbreviations: *UMFA* Unmetabolized folic acid.

Concentrations of UMFA in maternal plasma were significantly positively associated with human milk UMFA (β-coefficient = 0.5 nmol/L, 95% CI = 0.3 to 0.7 nmol/L); maternal plasma UMFA explained ~ 40% of the variability in human milk UMFA (pseudo R^2^ = 0.4); see Fig. 3.

## Discussion

This investigation provides evidence that the human milk folate profile can be altered by maternal folate supplementation; at a dose of 0.6 mg/day, folic acid resulted in significantly higher UMFA in human milk as compared to (6*S*)-5-MTHF. While 5-MTHF has historically been regarded the predominant folate form in human milk^6,11^, this may be shifting in the modern era where most pregnant women in Canada^33^ and the United States^34^ supplement with folic acid-containing prenatal multivitamins and consume folic acid fortified foods.

The effect of folic acid supplementation on human milk UMFA was fairly congruent with previous evidence, and further supports that the impact of maternal folic acid supplementation on the human milk folate profile is dose-dependent. In the current study, UMFA represented ~ 28% of total milk folate following supplementation with 0.6 mg/day folic acid; in other reported studies, UMFA represented ~ 8% of total milk folate following a lower dose (0.4 mg/day)^19^, and ~ 40–50% following higher doses (0.75 mg/day^20^ and > 0.4 mg/day^13^). Of note, participants of the MIREC cohort^13^ reported the dose of folic acid consumed in the past 30 days prior to milk sampling and were classified as consuming > 0.4 mg/day; it was later reported that most MIREC participants consumed 1.0 mg/day during pregnancy^35^, given available prenatal vitamins on the Canadian market. This may explain why the proportion of UMFA in those supplementing with > 0.4 mg/day was higher (~ 50%) as compared to the current findings at 0.6 mg/day (~ 28%) and West et al.^20^ at 0.75 mg/day (~ 40%). Timing of folic acid supplementation (2 h prior to milk collection) may have contributed to an increased UMFA transfer in the current study; following folic acid intake, UMFA increases in plasma, peaking after ~ 1–2 h, followed by clearance over the next several hours^36–38^. Maternal plasma UMFA and human milk UMFA were positively associated in the current study (pseudo R^2^ = 0.4). However, West et al.^20^ collected human milk specimens after an overnight fast, yet UMFA represented ~ 40% of total milk folate^20^. Ultimately, the impact of fasting state and other factors which influence the transfer of UMFA into human milk (perhaps concentrations of FBP) warrant further investigation.

Conversely, milk UMFA following (6*S*)-5-MTHF supplementation (~ 2% proportion) was lower than previous similar reports. In studies where participants reported no supplemental folate of any form, the proportion of UMFA was ~ 23% (MIREC)^13^ and ~ 18% (baseline; West et al.)^20^; in a randomized trial of 0.4 mg/day (6*S*)-5-MTHF or placebo, UMFA represented ~ 8% of total milk folate in both groups^19^. Reasons for higher human milk UMFA noted in previous studies as compared to the current trial are unknown, but may include differences in the study design (self-reported folic acid supplementation^13,20^ versus clinical trial participation^19^), the human milk collection methods, and assay used for quantification of folate forms. The current trial, MIREC cohort^13^, and West et al.^20^ utilized LC–MS/MS for the quantification of human milk folate forms, whereas ion-pair HPLC with electrochemical detection was used in the randomized trial by Houghton et al.^19^. Thus, differences in detectors may limit the ability to make comparisons across these studies; LC–MS/MS offers greater sensitivity over HPLC, as stable isotope labeled internal standards improve precision and accuracy^13,20,39,40^. Finally, if folic acid-containing foods were consumed habitually, particularly in the hours prior to the milk collection, it may have contributed to increased milk UMFA, despite no folic acid supplementation.

Previous evidence suggests that UMFA may be incorporated into human milk at the expense of 5-MTHF^13^. In the current trial, median milk 5-MTHF concentrations were 7.2 nmol/L lower in those supplementing with folic acid as compared to (6*S*)-5-MTHF, despite similar concentrations of maternal plasma 5-MTHF in each group (see Table 2); while the 95% CI for the difference in human milk 5-MTHF between groups crossed zero, non-significance may have been due to a lack of power, given that differences in crude estimates appear meaningful with minimal overlap in IQRs (see Table 2). The underlying mechanism may include a stronger affinity of FRα for UMFA as compared to reduced forms. It is worth noting that once UMFA is incorporated into human milk via binding to FBP, it likely cannot be cleared via mechanisms observed in plasma (e.g., metabolism to 5-MTHF). Ultimately, our study cannot ascertain whether UMFA was transferred preferentially into human milk over 5-MTHF; this phenomenon should be further explored via use of stable isotopes.

It has been hypothesized that UMFA in human milk could negatively impact infant folate status^13,41^. In vitro studies report that UMFA remains more tightly bound to soluble FBP (a cleavage product of the mammary epithelial-associated FRα) than 5-MTHF during gastric passage, reducing bioavailability of UMFA upon ingestion^41–43^. However, evidence from suckling rats note that the folic acid-FBP complex can attach at the intestinal brush-border for absorption without dissociation^44^. Further, the human intestine is permeable in early life, enabling the absorption of proteins^45^. Again, UMFA in adults is cleared from plasma via increased metabolism and excretion^46,47^; the rate at which this occurs in infants is not established, but UMFA has been detected in plasma and feces of breastfed infants^48,49^. Increased folate excretion due to higher ingested UMFA may be an alternative mechanism by which infant folate status is impacted.

Ultimately, the biological and clinical consequences of the capacity to absorb protein-bound folate and subsequent influx of UMFA into infant circulation remains unclear. Current human studies are mainly limited to observational designs and have produced mixed results for outcomes including neurological disorders and allergic diseases, and generally fail to demonstrate an association with UMFA specifically^50–59^. However, provision of excess maternal folic acid in animal models has been associated with altered 1C metabolism and adverse cognitive and metabolic outcomes of offspring^60–65^. It is feasible that continuous UMFA exposure while breastfeeding may influence folate-related metabolism via competition with reduced folates for transporters, binding proteins, and folate-related enzymes^21,28,66^. Further, it is hypothesized that the potential adverse effects of UMFA may be proportional to the level of exposure^21^. Elucidating the role of folic acid supplementation in pregnancy and UMFA in predisposition to future health and disease risk has been deemed a research priority by experts in the field of folate and perinatal nutrition^21,32^.

Strengths of this investigation include the rigorous methods for milk collection, adherence to the study protocol and daily supplementation, and quantification methods which captured total milk folate including 5-MTHF degradation product, MeFox. The association between UMFA in maternal plasma and human milk is limited by sample collection on separate days; however, it is unlikely that this substantially biased results, as human milk and blood collections occurred within ≤ 4 days for almost all participants, with standardized folate supplementation prior to collection. It is possible that the difference in milk UMFA between groups would have been less apparent if folate supplementation had not occurred 2 h prior to the milk collection; however, our findings represent the impact of folic acid consumption (via supplements or possibly fortified foods) on the human milk folate profile when maternal plasma UMFA may be at its peak. Finally, human milk folate concentrations increase with advancing lactation, with lowest concentrations in colostrum, peaking at 2–3 months postpartum^11,67,68^; thus, generalizability of our results (transition milk) to other studies examining human milk sampled at different stages (colostrum or mature milk) may be limited.

In conclusion, as human milk may serve as the sole source of infant nutrition, it is imperative to understand factors which impact its composition. While others have shown that supplemental folic acid at recommended doses (0.4 mg/day) did not increase concentrations of human milk UMFA as compared to (6*S*)-5-MTHF or a placebo^19^, we demonstrate that at higher doses (0.6 mg/day), there was a differential impact. While the downstream effects of elevated UMFA are unclear, current practices of folic acid supplementation may be shifting the “normal” human milk folate profile, given that commercially available prenatal multivitamins contain higher than recommended doses (0.6–1.0 mg). Our results suggest that reduction of UMFA in human milk may be effectively achieved by supplementation with (6*S*)-5-MTHF as opposed to synthetic folic acid. In the interim, re-evaluation of the human milk folate profile and infant folate status in the post-fortification era, and prospective monitoring to determine any association with adverse outcomes in childhood and beyond, should be prioritized.

## Methods

The full study protocol is published elsewhere^69^. Pregnant women (*n* = 60) in Vancouver, Canada, aged 19–42 years with singleton pregnancies were enrolled. Exclusion criteria included medical conditions, medications, and lifestyle factors associated with altered folate status or an increased risk of neural tube defects^70^. The study occurred between Sept/2019 to Sept/2021. Baseline visits took place at 8–21 weeks’ gestation; informed consent was obtained and demographic data and medical/nutrition history were reported. All participants reported folate supplementation prior to study enrollment (64% reporting commencement of supplementation preconceptionally). Dietary folate intake was assessed via a food frequency questionnaire (NutritionQuest, Berkeley, CA^71^). Participants were randomized to 0.6 mg/day folic acid or an equimolar dose (0.625 mg) of (6*S*)-5-MTHF; all participants also received a prenatal vitamin (NPN: 80025456) without folic acid, to ensure adequacy of other nutrients. Folate group allocations remained double-blinded until statistical analyses were complete.

The primary phase of the study included evaluation of folate status during pregnancy; participants had the option to continue to the postpartum study phase, which included obtaining separate informed consent at the endline visit (occurred 16-weeks after baseline) and continued supplementation until ~ 1 week postpartum for collection of a human milk and blood specimen. Adherence to daily folate supplementation throughout the study was assessed with capsule counts. The sample size was chosen for the primary study phase^69^. The trial is registered at ClinicalTrials.gov (NCT04022135) and was approved by the UBC Clinical Research Ethics Board (H18-02635). The study supplements were approved for clinical trial use by the natural and non-prescription health products directorate of Health Canada (Submission No. 244456). All methods were performed in accordance with the relevant guidelines and regulations.

### Human milk collection

At endline visits, participants were trained and provided with the required supplies to collect a human milk specimen in their home at day 5–7 postpartum. Instructions indicated collection from the right breast, between 1300–1450 h, at the start of a new feed (2–3 h after the last expression), and intake of the study supplements 2 h prior to collection^72^. Supplement intake timing was selected to standardize any peak in maternal plasma UMFA concentrations^36,38^, and reduce any subsequent bias on milk UMFA. Participants were instructed to fully express milk manually or by electric pump; however, if a partial expression was preferred, participants were instructed to express milk at the start and end of the feed. Participants were given an amber-colored collection jar to protect the labile folate from light, or if using an electric pump, tinfoil was provided to wrap the bottle. Once the milk was expressed, participants were instructed to gently swirl it, and then using a sterile plastic falcon pipette, 0.5 mL milk was transferred into two 2-mL amber cryovials (containing 0.005 g ascorbic acid for a 1% dilution). The vials were gently inverted and stored in a provided box in their household freezer (aiming for pick-up in ≤ 4 days); frozen milk samples were transported to the laboratory for storage at -80 °C.

### Maternal blood specimen collection and processing

Upon human milk specimen pick-up, participants opted to provide a non-fasting blood specimen for plasma UMFA and 5-MTHF analysis, collected in a 6-mL EDTA tube (BD Biosciences, Franklin Lakes, NJ). To match conditions at the human milk collection, participants were instructed to consume their folate supplements 2 h prior to the blood draw. Vacutainers were shielded from light, inverted gently, and transported to the laboratory for immediate processing (within 2 h of collection). Vacutainers were centrifuged at 3000 rpm × 15 min at 4 °C. Plasma was aliquoted and stored at -80 °C.

### Quantification of folate forms

Human milk folate forms (nmol/L), including UMFA, 5-MTHF, tetrahydrofolate (THF), 5,10-methenylTHF, 5-formylTHF, and 4α-hydroxy-5-MTHF (MeFox; oxidation product of 5-MTHF), and maternal plasma UMFA (nmol/L) and plasma 5-MTHF (nmol/L), were determined via liquid chromatography-tandem mass spectrometry (LC-MS/MS); detailed description of quantification methods are published elsewhere^13,40,73,74^. Total milk folate (nmol/L) equalled the sum of all folate forms (including MeFox). Inter-assay variability of each folate form was determined using internal quality control samples; the coefficient of variation ranged from 3 to 6% for human milk folates and was 8% for plasma UMFA and 4% for plasma 5-MTHF.

### Statistical analyses

The effect of folic acid versus (6*S*)-5-MTHF on human milk folate forms and total folate concentrations, adjusting for dietary folate intake, total weeks of supplementing, and adherence to the human milk collection protocol (a full or partial milk expression, as there were differences between groups), was evaluated with multivariable linear (total folate) or quantile (folate forms) regression. Normality of the data was evaluated via use of skewness and kurtosis tests. Quantile regression was chosen over data transformations to account for the non-normal distribution of the human milk folate forms, as it leads to more straightforward interpretations of the model estimates^75^. Covariates were chosen a priori^69^. Concentrations of UMFA in human milk and plasma in each group were pooled and quantile regression was used to examine their association; pooling of data from both groups was chosen to examine the overall association, while preserving the largest sample size possible. All analyses were completed on an intention-to-treat basis. A two-tailed *P* < 0.05 was considered statistically significant.

## Supplementary Information

Supplementary Information.

## Data Availability

The datasets generated during and/or analysed during the current study are available from the corresponding author on reasonable request.
