# Supplementary material for: Human milk unmetabolized folic acid is increased following supplementation with synthetic folic acid as compared to (6S)-5-methyltetrahydrofolic acid
Source: Sci Rep. 2023 Jul 12;13:11298. doi: 10.1038/s41598-023-38224-4 (PMC10338559; doi:10.1038/s41598-023-38224-4)
Supplement: Supplementary file 1 — Supplementary Information. [file 41598_2023_38224_MOESM1_ESM.pdf]

Cochrane, KM, et al. Human milk unmetabolized folic acid is increased following supplementation with synthetic folic acid as compared to (6*S*)-5-methyltetrahydrofolic acid

Supplemental Table 1: *Human milk folate forms and total milk folate among those supplemented with (6S)-5-MTHF (n=22) or folic acid (n=21) (Vancouver, Canada, 2019-2021)*

Human milk folate form	β-coefficient (95% CI)
**UMFA (nmol/L)**	
Supplemental folate group (folic acid)	11 (6.4, 17)
Milk expression (full expression)	-0.6 (-6, 4.9)
Total weeks of supplementing	0.02 (-0.6, 0.6)
Dietary folate (mg DFE/day)	-0.9 (-17, 15)
**5-MTHF (nmol/L)**	
Supplemental folate group (folic acid)	-9 (-20, 1.8)
Milk expression (full expression)	2.8 (-8.7, 14)
Total weeks of supplementing	0.5 (-0.7, 1.7)
Dietary folate (mg DFE/day)	0.4 (-33, 34)
**THF (nmol/L)**	
Supplemental folate group (folic acid)	-0.8 (-9.4, 7.9)
Milk expression (full expression)	1.3 (-8, 11)
Total weeks of supplementing	0.5 (-0.5, 1.5)
Dietary folate (mg DFE/day)	3.5 (-23, 30)
**5,10-methenylTHF (nmol/L)**	
Supplemental folate group (folic acid)	-0.6 (-1.8, 0.6)
Milk expression (full expression)	-0.3 (-1.6, 1)
Total weeks of supplementing	0.04 (-0.1, 0.2)
Dietary folate (mg DFE/day)	1.4 (-2.4, 5.1)
**5-formylTHF (nmol/L)**	
Supplemental folate group (folic acid)	0.3 (-0.2, 0.8)
Milk expression (full expression)	-0.2 (-0.7, 0.4)
Total weeks of supplementing	0.04 (-0.01, 0.1)
Dietary folate (mg DFE/day)	0.7 (-0.8, 2.2)
**MeFox (nmol/L)**	
Supplemental folate group (folic acid)	0.07 (-0.1, 0.3)
Milk expression (full expression)	-0.1 (-0.3, 0.07)
Total weeks of supplementing	0.02 (-0.002, 0.04)
Dietary folate (mg DFE/day)	0.6 (0.04, 1.3)
**[1]Total folate (nmol/L)**	
Supplemental folate group (folic acid)	13 (-2.9, 29)
Milk expression (full expression)	-0.8 (-18, 16)
Total weeks of supplementing	1.9 (0.09, 3.8)
Dietary folate (mg DFE/day)	4.1 (-46, 54)

n=43 Milk folate forms and total folate adjusted for: supplemental folate form ([6*S*]-5-MTHF as the reference group), milk expression (partial expression as the reference group), total weeks of supplementing, dietary folate intake (mg dietary folate equivalents/d). [1]Total folate = sum of all forms. Abbreviations: 5-MTHF: 5-methyltetrahydrofolate; DFE: Dietary folate equivalents; UMFA: unmetabolized folic acid; THF: tetrahydrofolate.
